# 4-Amino-3-(4-hy­droxy­benz­yl)-1*H*-1,2,4-triazole-5(4*H*)-thione

**DOI:** 10.1107/S1600536813033370

**Published:** 2013-12-14

**Authors:** B. K. Sarojini, P. S. Manjula, Manpreet Kaur, Brian J. Anderson, Jerry P. Jasinski

**Affiliations:** a Department of Chemistry, P.A. College of Engineering, Nadupadavu 574 153, D.K., Mangalore, India; b Department of Studies in Chemistry, Mangalore University, Mangalagangotri 574 199, Mangalore, India; c Department of Studies in Chemistry, University of Mysore, Manasagangotri, Mysore 570 006, India; d Department of Chemistry, Keene State College, 229 Main Street, Keene, NH 03435-2001, USA

## Abstract

In the title compound, C_9_H_10_N_4_OS, the dihedral angle between the benzene and 1*H*-1,2,4-triazole-5(4*H*)-thione rings is 67.51 (16)°. In the crystal, mol­ecules are liked *via* N—H⋯O hydrogen bonds, forming chains along the *c*-axis direction. The chains are linked *via* O—H⋯S hydrogen bonds, forming corrugated layers lying parallel to the *bc* plane. The layers are linked *via* N—H⋯N and N—H⋯S hydrogen bonds, forming a three-dimensional network.

## Related literature   

For biological properties of 1,2,4-triazole derivatives, see: Holla *et al.* (2001 ▶, 2006 ▶); Mullican *et al.* (1993 ▶); Jones *et al.* (1965 ▶); Shams El-Dine *et al.* (1974 ▶); Misato *et al.* (1977 ▶); Kane *et al.* (1988 ▶). For related structures, see: Puviarasan *et al.* (1999 ▶); Chen *et al.* (2007 ▶); Karczmarzyk *et al.* (2012 ▶); Gao *et al.* (2011 ▶).
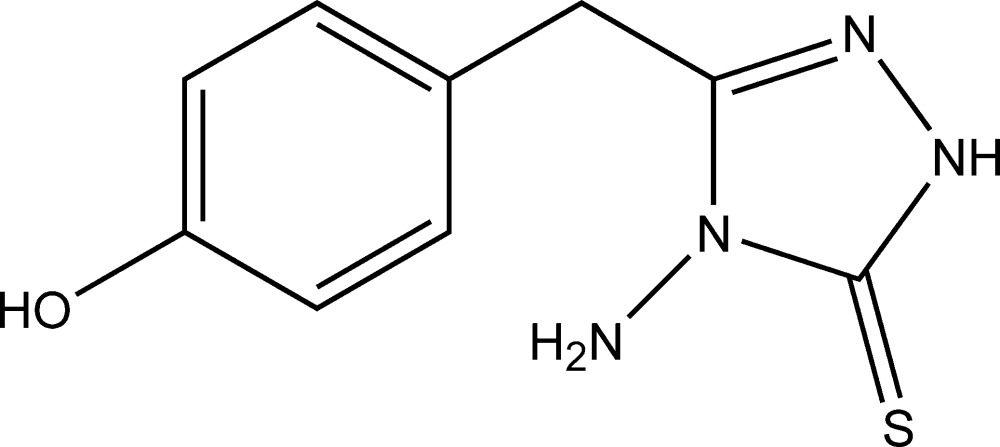


## Experimental   

### 

#### Crystal data  

C_9_H_10_N_4_OS
*M* *_r_* = 222.27
Triclinic, 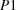

*a* = 4.2117 (5) Å
*b* = 6.1891 (7) Å
*c* = 10.0641 (11) Å
α = 100.590 (9)°
β = 94.916 (9)°
γ = 104.589 (10)°
*V* = 247.14 (5) Å^3^
*Z* = 1
Mo *K*α radiation
μ = 0.31 mm^−1^
*T* = 173 K
0.34 × 0.30 × 0.24 mm

#### Data collection  

Agilent Gemini EOS diffractometer
Absorption correction: multi-scan (*CrysAlis PRO* and *CrysAlis RED*; Agilent, 2012 ▶). *T*_min_ = 0.941, *T*_max_ = 1.000
2555 measured reflections
1897 independent reflections
1826 reflections with *I* > 2σ(*I*)
*R*_int_ = 0.035

#### Refinement  

*R*[*F* ^2^ > 2σ(*F* ^2^)] = 0.041
*wR*(*F* ^2^) = 0.102
*S* = 1.10
1897 reflections
145 parameters
3 restraints
H atoms treated by a mixture of independent and constrained refinement
Δρ_max_ = 0.29 e Å^−3^
Δρ_min_ = −0.27 e Å^−3^
Absolute structure: Flack (1983 ▶), 265 Friedel pairs (15% coverage)
Absolute structure parameter: −0.02 (9)

### 

Data collection: *CrysAlis PRO* (Agilent, 2012 ▶); cell refinement: *CrysAlis PRO*; data reduction: *CrysAlis RED* (Agilent, 2012 ▶); program(s) used to solve structure: *SUPERFLIP* (Palatinus & Chapuis, 2007 ▶); program(s) used to refine structure: *SHELXL2012* (Sheldrick, 2008 ▶); molecular graphics: *OLEX2* (Dolomanov *et al.*, 2009 ▶); software used to prepare material for publication: *OLEX2*.

## Supplementary Material

Crystal structure: contains datablock(s) global, I. DOI: 10.1107/S1600536813033370/su2674sup1.cif

Structure factors: contains datablock(s) I. DOI: 10.1107/S1600536813033370/su2674Isup2.hkl

Supporting information file. DOI: 10.1107/S1600536813033370/su2674Isup3.cml

Additional supporting information:  crystallographic information; 3D view; checkCIF report

## Figures and Tables

**Table 1:** Hydrogen-bond geometry (Å, °)

*D*—H⋯*A*	*D*—H	H⋯*A*	*D*⋯*A*	*D*—H⋯*A*
N2—H2⋯O1^i^	0.88	1.97	2.840 (3)	170
O1—H1⋯S1^ii^	0.95 (5)	2.27 (5)	3.180 (2)	159 (4)
N4—H4*A*⋯S1^iii^	0.86	2.74	3.435 (3)	138
N4—H4*B*⋯N1^iv^	0.91 (3)	2.32 (3)	3.149 (4)	153 (3)

Symmetry codes: (i) 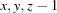; (ii) 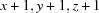; (iii) 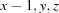; (iv) 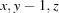.

**Fig. 1.**
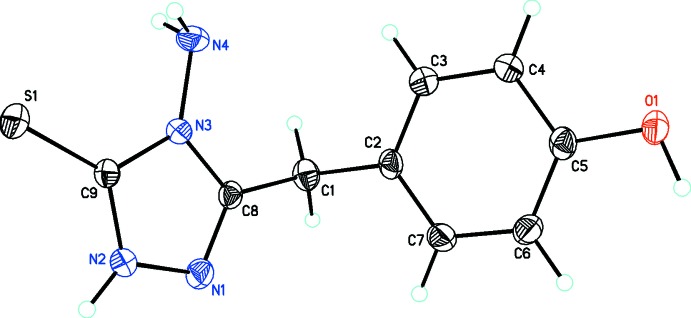
A view of the molecular structure of the title molecule, with atom labelling. The displacement ellipsoids are drawn at the 50% probability level.

**Fig. 2.**
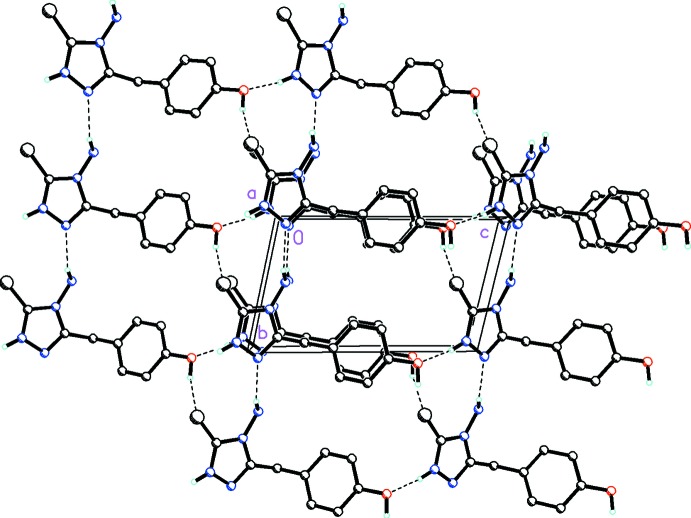
A view along the a axis of the crystal packing of the title compound. The hydrogen bonds are shown as dashed lines; see Table 1 for details; H atoms not involved in hydrogen bonding have been omitted for clarity.

**Fig. 3.**
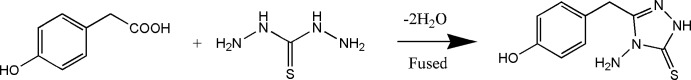
Synthesis of the title compound.

## supplementary crystallographic information

### 1. Comment

The chemistry of triazoles has received considerable attention in recent years because of their versatility in the synthesis of many other heterocyclic compounds. 1,2,4-Triazole derivatives are well known for their different biological activities, therefore various 1,2,4-triazole derivatives and their N-bridged heterocyclic analogs have been extensively studied (Holla *et al.*, 2001,2006). The derivatives of 1,2,4-triazole are known to exhibit anti-inflammatory (Mullican *et al.*, 1993), antiviral (Jones *et al.*, 1965), antimicrobial (Shams El-Dine *et al.*, 1974; Misato *et al.*, 1977) and antidepressant activity (Kane *et al.*, 1988). Hence synthesis of the corresponding heterocyclic compounds could be of interest from the viewpoint of chemical reactivity and biological activity.

The crystal structures of some related triazoles have been reported: 5-(2-Chlorophenyl)-4-phenyl-3,4-dihydro-2*H*-1,2,4-triazole-3-thione (Puviarasan *et al.*, 1999); 4-Amino-5-(2-ethoxyphenyl)-2,4-dihydro- 2*H*-1,2,4-triazole-3-thione-triphenylphosphine oxide (Chen *et al.*, 2007); Ethyl2-(3-methyl-5-sulfanylidene-4,5-dihydro- 1*H*-1,2,4-triazol-4-yl)acetate (Karczmarzyk *et al.*, 2012); 3-(4-Amino-3-phenyl-5-sulfanylidene-4,5-dihydro-1*H*-1,2,4-triazol-1- yl)-3-(2-chlorophenyl)-1-phenylpropan-1-one (Gao *et al.*, 2011). The present work describes the synthesis and crystal structure of the title compound.

In the title compound, Fig. 1, the dihedral angle between the benzene ring (C2-C7) and the 1*H*-1,2,4-triazole-5(4*H*)-thione ring (N1/N2/C9/N3/C8) is 67.51 (16) °.

In the crystal, a single N2—H2···O1 hydrogen bond and additional weak O1—H1···S1, N4—H4A···S1 and N4—H4B···N1 hydrogen bonds are observed (Table 1 and Fig. 2). These interactions link the molecules into one-dimensional chains extending along each of the three axes forming a three-dimensional supramolecular framework.

### 2. Experimental

The synthesis of the title compound is described in Fig. 3. A well triturated mixture of 4-hydroxyphenylacetic acid (0.755 g, 0.005 mol) and thiocarbohydrazide (0.53 g, 0.005 mol) was fused in a round bottom flask for one hour on an oil bath at 413 K. It was cooled to room temperature and washed with sodium bicarbonate (5%) solution to remove unreacted acid and again washed with water. The dried compound was recrystallized from methanol yielding colourless block-like crystals (M.p. 475-477 K).

### 3. Refinement

The OH and NH_2_ H atoms (H1, and H4A/H4B, respectively) were located in a difference Fourier map and freely refined. The remaining H atoms were placed in calculated positions and refined using the riding model approximation: N-H = 0.88 Å, C-H = 0.95 and 0.99 Å for CH and CH_2_ H atoms, respectively, with U_iso_(H) = 1.2U_eq_(N,C).

### Crystal data

**Table:**

C_9_H_10_N_4_OS	*Z* = 1
*M**_r_* = 222.27	*F*(000) = 116
Triclinic, *P*1	*D*_x_ = 1.493 Mg m^−^^3^
*a* = 4.2117 (5) Å	Mo *K*α radiation, λ = 0.71073 Å
*b* = 6.1891 (7) Å	Cell parameters from 1329 reflections
*c* = 10.0641 (11) Å	θ = 3.5–32.8°
α = 100.590 (9)°	µ = 0.31 mm^−^^1^
β = 94.916 (9)°	*T* = 173 K
γ = 104.589 (10)°	Block, colourless
*V* = 247.14 (5) Å^3^	0.34 × 0.30 × 0.24 mm

### Data collection

**Table:**

Agilent Gemini EOS diffractometer	1897 independent reflections
Radiation source: Enhance (Mo) X-ray Source	1826 reflections with *I* > 2σ(*I*)
Detector resolution: 16.0416 pixels mm^-1^	*R*_int_ = 0.035
ω scans	θ_max_ = 32.9°, θ_min_ = 3.5°
Absorption correction: multi-scan (*CrysAlis PRO* and *CrysAlis RED*; Agilent, 2012).	*h* = −6→6
*T*_min_ = 0.941, *T*_max_ = 1.000	*k* = −8→9
2555 measured reflections	*l* = −14→12

### Refinement

**Table:**

Refinement on *F*^2^	Hydrogen site location: mixed
Least-squares matrix: full	H atoms treated by a mixture of independent and constrained refinement
*R*[*F*^2^ > 2σ(*F*^2^)] = 0.041	*w* = 1/[σ^2^(*F*_o_^2^) + (0.0512*P*)^2^] where *P* = (*F*_o_^2^ + 2*F*_c_^2^)/3
*wR*(*F*^2^) = 0.102	(Δ/σ)_max_ < 0.001
*S* = 1.10	Δρ_max_ = 0.29 e Å^−^^3^
1897 reflections	Δρ_min_ = −0.27 e Å^−^^3^
145 parameters	Absolute structure: Flack (1983), 265 Friedel pairs (15% coverage)
3 restraints	Absolute structure parameter: −0.02 (9)
Primary atom site location: structure-invariant direct methods	

### Special details

**Table:**

Geometry. All esds (except the esd in the dihedral angle between two l.s. planes) are estimated using the full covariance matrix. The cell esds are taken into account individually in the estimation of esds in distances, angles and torsion angles; correlations between esds in cell parameters are only used when they are defined by crystal symmetry. An approximate (isotropic) treatment of cell esds is used for estimating esds involving l.s. planes.

### Fractional atomic coordinates and isotropic or equivalent isotropic displacement parameters (Å^2^)

**Table:**

	*x*	*y*	*z*	*U*_iso_*/*U*_eq_	
S1	0.98393 (10)	0.49047 (8)	−0.16110 (7)	0.02134 (16)	
O1	1.3407 (6)	1.0709 (3)	0.7411 (2)	0.0248 (4)	
N1	0.8426 (6)	1.0486 (4)	0.0472 (3)	0.0217 (5)	
N2	0.9604 (6)	0.9245 (4)	−0.0560 (2)	0.0205 (5)	
H2	1.0738	0.9862	−0.1156	0.025*	
N3	0.7176 (5)	0.6844 (3)	0.0529 (2)	0.0164 (4)	
N4	0.5720 (6)	0.4828 (4)	0.0952 (3)	0.0227 (5)	
H4A	0.3813	0.4158	0.0464	0.027*	
C1	0.5336 (7)	0.9471 (5)	0.2357 (3)	0.0211 (5)	
H1A	0.3342	0.8192	0.2314	0.025*	
H1B	0.4603	1.0871	0.2357	0.025*	
C2	0.7593 (7)	0.9795 (4)	0.3678 (3)	0.0187 (5)	
C3	0.7404 (8)	0.8033 (5)	0.4367 (3)	0.0238 (6)	
H3	0.5876	0.6587	0.3991	0.029*	
C4	0.9400 (8)	0.8336 (5)	0.5596 (3)	0.0248 (6)	
H4	0.9254	0.7104	0.6049	0.030*	
C5	1.1611 (7)	1.0458 (5)	0.6157 (3)	0.0200 (5)	
C6	1.1905 (7)	1.2236 (5)	0.5479 (3)	0.0229 (6)	
H6	1.3466	1.3672	0.5849	0.027*	
C7	0.9885 (8)	1.1898 (5)	0.4243 (3)	0.0219 (5)	
H7	1.0071	1.3119	0.3779	0.026*	
C8	0.6955 (6)	0.8986 (4)	0.1125 (3)	0.0167 (5)	
C9	0.8850 (6)	0.7017 (4)	−0.0565 (3)	0.0168 (5)	
H1	1.500 (12)	1.216 (7)	0.758 (5)	0.038 (11)*	
H4B	0.713 (8)	0.395 (5)	0.079 (3)	0.009 (7)*	

### Atomic displacement parameters (Å^2^)

**Table:**

	*U*^11^	*U*^22^	*U*^33^	*U*^12^	*U*^13^	*U*^23^
S1	0.0238 (3)	0.0209 (3)	0.0187 (3)	0.0076 (2)	0.0035 (2)	0.0003 (2)
O1	0.0279 (10)	0.0253 (10)	0.0156 (10)	−0.0007 (8)	−0.0003 (8)	0.0027 (8)
N1	0.0283 (12)	0.0185 (10)	0.0187 (11)	0.0085 (9)	0.0033 (9)	0.0020 (8)
N2	0.0267 (12)	0.0182 (10)	0.0158 (11)	0.0041 (9)	0.0051 (9)	0.0039 (8)
N3	0.0182 (10)	0.0157 (9)	0.0148 (10)	0.0033 (8)	0.0018 (8)	0.0041 (8)
N4	0.0253 (11)	0.0180 (10)	0.0247 (13)	0.0021 (9)	0.0074 (10)	0.0079 (9)
C1	0.0215 (11)	0.0252 (12)	0.0185 (13)	0.0102 (10)	0.0038 (10)	0.0038 (10)
C2	0.0221 (11)	0.0212 (11)	0.0132 (12)	0.0070 (9)	0.0053 (9)	0.0017 (9)
C3	0.0278 (13)	0.0204 (11)	0.0203 (13)	0.0018 (10)	0.0023 (11)	0.0043 (10)
C4	0.0322 (14)	0.0202 (12)	0.0194 (13)	0.0012 (10)	0.0023 (11)	0.0061 (10)
C5	0.0215 (12)	0.0234 (12)	0.0145 (11)	0.0059 (10)	0.0058 (10)	0.0016 (9)
C6	0.0253 (13)	0.0194 (11)	0.0199 (13)	0.0007 (10)	0.0050 (11)	0.0008 (10)
C7	0.0289 (13)	0.0193 (11)	0.0187 (13)	0.0067 (10)	0.0067 (11)	0.0051 (9)
C8	0.0185 (11)	0.0173 (11)	0.0138 (11)	0.0059 (9)	−0.0013 (9)	0.0020 (9)
C9	0.0182 (11)	0.0173 (10)	0.0135 (11)	0.0034 (9)	0.0002 (9)	0.0026 (9)

### Geometric parameters (Å, º)

**Table:**

S1—C9	1.683 (3)	C1—H1B	0.9900
O1—C5	1.376 (4)	C1—C2	1.517 (4)
O1—H1	0.95 (4)	C1—C8	1.484 (4)
N1—N2	1.379 (3)	C2—C3	1.385 (4)
N1—C8	1.297 (4)	C2—C7	1.396 (4)
N2—H2	0.8800	C3—H3	0.9500
N2—C9	1.334 (3)	C3—C4	1.389 (4)
N3—N4	1.403 (3)	C4—H4	0.9500
N3—C8	1.380 (3)	C4—C5	1.390 (4)
N3—C9	1.364 (3)	C5—C6	1.384 (4)
N4—H4A	0.8645	C6—H6	0.9500
N4—H4B	0.91 (3)	C6—C7	1.395 (4)
C1—H1A	0.9900	C7—H7	0.9500
			
C5—O1—H1	106 (3)	C2—C3—C4	121.4 (3)
C8—N1—N2	104.5 (2)	C4—C3—H3	119.3
N1—N2—H2	123.4	C3—C4—H4	120.3
C9—N2—N1	113.2 (2)	C3—C4—C5	119.4 (3)
C9—N2—H2	123.4	C5—C4—H4	120.3
C8—N3—N4	124.5 (2)	O1—C5—C4	117.3 (3)
C9—N3—N4	126.6 (2)	O1—C5—C6	122.3 (2)
C9—N3—C8	108.8 (2)	C6—C5—C4	120.4 (3)
N3—N4—H4A	109.6	C5—C6—H6	120.4
N3—N4—H4B	104 (2)	C5—C6—C7	119.3 (3)
H4A—N4—H4B	109.9	C7—C6—H6	120.4
H1A—C1—H1B	107.8	C2—C7—H7	119.4
C2—C1—H1A	109.0	C6—C7—C2	121.1 (3)
C2—C1—H1B	109.0	C6—C7—H7	119.4
C8—C1—H1A	109.0	N1—C8—N3	110.1 (2)
C8—C1—H1B	109.0	N1—C8—C1	125.8 (2)
C8—C1—C2	113.0 (2)	N3—C8—C1	124.2 (2)
C3—C2—C1	121.1 (2)	N2—C9—S1	129.2 (2)
C3—C2—C7	118.3 (3)	N2—C9—N3	103.4 (2)
C7—C2—C1	120.6 (2)	N3—C9—S1	127.36 (19)
C2—C3—H3	119.3		
			
O1—C5—C6—C7	−176.7 (3)	C3—C2—C7—C6	−0.9 (4)
N1—N2—C9—S1	−178.7 (2)	C3—C4—C5—O1	176.6 (3)
N1—N2—C9—N3	−1.2 (3)	C3—C4—C5—C6	−2.1 (5)
N2—N1—C8—N3	−0.1 (3)	C4—C5—C6—C7	1.9 (4)
N2—N1—C8—C1	178.4 (3)	C5—C6—C7—C2	−0.4 (4)
N4—N3—C8—N1	−177.2 (3)	C7—C2—C3—C4	0.7 (4)
N4—N3—C8—C1	4.3 (4)	C8—N1—N2—C9	0.8 (3)
N4—N3—C9—S1	−4.9 (4)	C8—N3—C9—S1	178.64 (19)
N4—N3—C9—N2	177.5 (3)	C8—N3—C9—N2	1.1 (3)
C1—C2—C3—C4	−178.2 (3)	C8—C1—C2—C3	−98.8 (3)
C1—C2—C7—C6	178.1 (3)	C8—C1—C2—C7	82.2 (3)
C2—C1—C8—N1	−94.8 (3)	C9—N3—C8—N1	−0.7 (3)
C2—C1—C8—N3	83.5 (3)	C9—N3—C8—C1	−179.1 (2)
C2—C3—C4—C5	0.7 (5)		

### Hydrogen-bond geometry (Å, º)

**Table:**

*D*—H···*A*	*D*—H	H···*A*	*D*···*A*	*D*—H···*A*
N2—H2···O1^i^	0.88	1.97	2.840 (3)	170
O1—H1···S1^ii^	0.95 (5)	2.27 (5)	3.180 (2)	159 (4)
N4—H4*A*···S1^iii^	0.86	2.74	3.435 (3)	138
N4—H4*B*···N1^iv^	0.91 (3)	2.32 (3)	3.149 (4)	153 (3)

Symmetry codes: (i) *x*, *y*, *z*−1; (ii) *x*+1, *y*+1, *z*+1; (iii) *x*−1, *y*, *z*; (iv) *x*, *y*−1, *z*.
